# Multiparametric Investigation of Dynamics in Fetal Heart Rate Signals

**DOI:** 10.3390/bioengineering9010008

**Published:** 2021-12-28

**Authors:** Alfonso Maria Ponsiglione, Francesco Amato, Maria Romano

**Affiliations:** Department of Electrical Engineering and Information Technology (DIETI), University of Naples “Federico II”, 80125 Naples, Italy; alfonsomaria.ponsiglione@unina.it (A.M.P.); framato@unina.it (F.A.)

**Keywords:** fetal heart rate variability, biomedical signal processing and analysis, cardiotocography, artificial neural networks, accelerations

## Abstract

In the field of electronic fetal health monitoring, computerized analysis of fetal heart rate (FHR) signals has emerged as a valid decision-support tool in the assessment of fetal wellbeing. Despite the availability of several approaches to analyze the variability of FHR signals (namely the FHRV), there are still shadows hindering a comprehensive understanding of how linear and nonlinear dynamics are involved in the control of the fetal heart rhythm. In this study, we propose a straightforward processing and modeling route for a deeper understanding of the relationships between the characteristics of the FHR signal. A multiparametric modeling and investigation of the factors influencing the FHR accelerations, chosen as major indicator of fetal wellbeing, is carried out by means of linear and nonlinear techniques, blockwise dimension reduction, and artificial neural networks. The obtained results show that linear features are more influential compared to nonlinear ones in the modeling of HRV in healthy fetuses. In addition, the results suggest that the investigation of nonlinear dynamics and the use of predictive tools in the field of FHRV should be undertaken carefully and limited to defined pregnancy periods and FHR mean values to provide interpretable and reliable information to clinicians and researchers.

## 1. Introduction

As widely showed in the literature concerning heart rate variability [1,2,3,4], computerized signal processing techniques proved to be an effective way to detect different types of cardiovascular disorders. Indeed, the processing and computerized analysis of heart rate signals, with particular regard to the heart rate variability (HRV), contributed to enhance the diagnostic value brought by the clinical diagnostic techniques, by providing additional information and data that can support the clinical decision-making and leading to standardized, reliable, and early diagnoses [5,6,7,8,9,10,11,12,13].

Computerized tools have been also applied to the analysis of the fetal heart rate (FHR) in order to assess those parameters that are not evaluable by the naked eye, such as the variability of the FHR (FHRV), which has been demonstrated to be a crucial parameter in the study of functional states and development of the autonomic nervous system of the fetus over the gestational age [14,15,16]. While cardiotocography (CTG) remains the gold standard in clinical practice, due to its widespread employment and legal value in some countries, for the recording of FHR and uterine contractions signals, assessment of FHR traces, and its FHRV are considered relevant factors in fetal wellbeing evaluation, there is still a lack of agreement within both the medical and the scientific community on a reference methodology for the analysis and interpretation of the FHRV, as well as the classification of CTG recordings [17,18,19].

The efforts towards the establishment of a standard technique for the processing and analysis of FHR signals brought the development of different software solutions, methodological approaches, and indicators that could assist the clinical examination of CTG recordings, with particular regard to the FHR signals [18,19,20,21,22,23,24]. As also happened in the analysis of adult and newborn heart rate signals [25,26,27,28], most of the newer computerized tools for FHR processing and analysis are based on Artificial Intelligence (AI) algorithms aimed at extracting novel features from the FHR signals, and achieve a more accurate classification of the traces according to the fetal health status [29,30,31,32]. Among the proposed tools, machine learning algorithms and, in particular, Artificial Neural Networks (ANN) showed promising results in terms of predictability and classification capabilities [31,32,33,34,35,36].

The value these novel techniques can bring mainly relies on the opportunity to enhance the diagnostic power of the current analytic methods. However, despite the wide availability of applications in this field, not all the studies deeply investigate the role and influence that such advanced tools could have in the interpretation of heart rate dynamics, and in expanding the current knowledge on the physiological mechanisms underlying the FHRV.

In this context, in this paper we propose a multiparametric approach to investigate the relationships between some of the most recognized FHRV features and the effect that they have on the accelerations of the heart rhythm, which are a well-known indicator of a healthy fetal status. Indeed, a reassuring FHR trace is mainly evaluated on the basis of the presence of normal variability and accelerations of the heartbeat, which are associated with fetal movements and wellbeing while non-reassuring traces are often characterized by low variability and absence of accelerations [37,38,39,40].

In particular, considering the primary role of both FHRV and accelerations in establishing the fetal activity and wellbeing from CTG recordings, here we propose a method to take advantage of linear and nonlinear indices of FHRV, and build a regression model to study the impact that these indices, with particular attention to nonlinear ones, have on the number of accelerations in FHR traces.

The proposed study provides an easy-to-design and straightforward biosignal processing and modeling route that could lead to a deeper understanding of the relationships between the major characteristics of the FHR signals.

## 2. Materials and Methods

### 2.1. Dataset

The examined dataset included 580 FHR signals recorded from healthy pregnant women, who had taken no drugs, both in public and private hospitals, by using available clinical CTG equipment. The signals were acquired using HP-135x or Sonicaid CTG, both equipped with an ultrasound Doppler probe to detect the FHR signal and an external pressure transducer to record the uterine contraction signal. A serial number identifies the signals. CTG were recorded from women between 28th and 42nd pregnancy week. Other information, such as Apgar score, birth weight, or associated pathologies, were gathered for further processing, when available. Twin pregnancies were not involved in the study. Gestational age was determined by physicians from the last menstruation date or from ultrasound measurements.

### 2.2. Methodological Workflow

**Figure 1:**
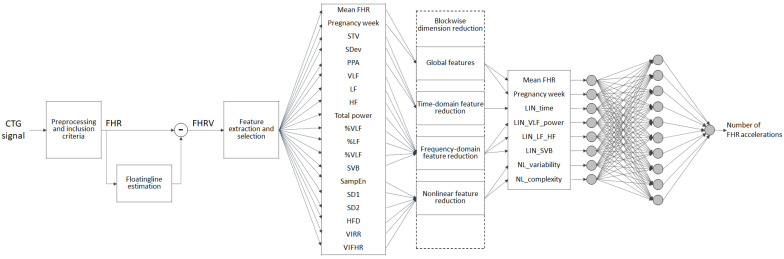
Methodological workflow. (STV = Short Term Variability; SDev = Standard Deviation; PPA = peak-to-peak amplitude; VLF and %VLF = power and its percentage in the Very Low Frequency band; LF and %LF = power and its percentage in the Low Frequency band; HF and %HF = power and its percentage in the High Frequency band; SVB = Sympatho-Vagal Balance; SampEn = Sample Entropy; SD1 = Standard Deviation 1 perpendicular to the line-of-identity obtained by Poincaré maps; SD2 = Standard Deviation 2 along the line-of-identity obtained by Poincaré maps; HFD = Higouchi Fractal Dimension; VIRR = Variability Index obtained by applying the Symbolic Dynamics technique to the RR time series; VIFHR = Variability Index obtained by applying the Symbolic Dynamics technique to the FHRV signal).

The methodological workflow, as depicted in Figure 1, illustrates the major steps adopted in the processing and analysis of the CTG signals, going from the preprocessing phase to the features extraction and selection, the dimension reduction, and the ANN analysis.

Each of the steps displayed in Figure 1 are described in detail in the following paragraphs, with particular regard to the blockwise dimension reduction step, which has been introduced in order to enhance the interpretability of the obtained ANN results.

### 2.3. Preprocessing, Inclusion Criteria, and Estimation of the FHRV Signal

The FHR signal recorded by the CTG technique was pre-processed with the main aim of detecting and managing outliers and signal loss (within 3 s duration), which can compromise the estimation of the FHRV in accordance with [41,42]. In compliance with the clinical practice, from the whole dataset of 580 traces, a subset of 187 signals were included in the analysis based on the following quality criteria:

- Absence of visually evident artifacts (determined by visual inspection of the signals);
- Absence of prolonged signal loss (i.e., higher than 30% signal length);
- Limited number of outliers (less than 5%);
- Minimum duration of the signal equal to 20 min.

**Figure 2:**
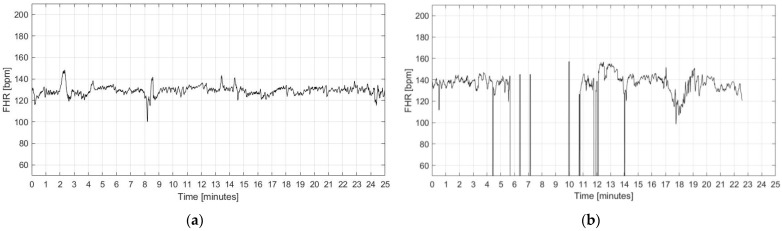
Examples of FHR signals: (**a**) included in the analysis; (**b**) excluded from the analysis due to the presence of artifacts and signal loss.

Examples of included and excluded signals are reported in Figure 2.

**Figure 3:**
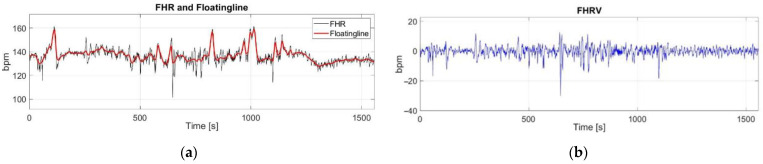
FHRV estimation: (**a**) FHR signal with floatingline (superimposed in red); (**b**) estimated FHRV.

The FHRV signal was estimated following a methodology previously described by the authors [20,43]. The floatingline, which can be defined as an imaginary line which follows slow alterations of fetal heart rate, is assessed by applying a nonlinear filtering with a varying smoothing parameter. The floatingline is then subtracted from the FHR signal in order to achieve an estimation of the FHRV (Figure 3).

### 2.4. Feature Extraction and Selection

Linear features of the FHRV were extracted by carrying out both time- and frequency-domain analyses of the FHRV signal. Nonlinear features were extracted by employing different nonlinear methodologies to the analysis of the FHRV.

The selected features are summarized in the following:

Two global features related to the patients’ clinical data and to the FHR signal were included as general descriptors of the processed signal:

- the week of gestation (estimated from the last menstruation);
- the mean value of the heart rate.

Three time-domain features were calculated as temporal and statistical parameters of the FHRV signal, as reported in previous publications [44,45]:

- Short Term Variability (STV), assessed as the mean of the standard deviation calculated on a 30 s sliding window on the FHRV, obtained after subtraction of the floatingline from the FHR signal, as already reported in [19,44];
- Standard Deviation of the FHRV (SDev), calculated as the standard deviation of the whole FHRV signal, obtained after subtraction of the floatingline from the FHR signal;
- Peak-to-Peak amplitude (PPA), calculated on the FHR signal.

Eight frequency-domain indices computed by estimating the Power Spectral Density through the Short Time Fourier Transform of the FHRV signal, as outlined in previous studies of the authors [45,46,47]:

- Power and percentages (with respect to the total power) in the very low frequency band (VLF and %VLF), low frequency band (LF and %LF), and high frequency band (HF and %HF); respectively defined in the following frequency ranges: VLF = 0–0.003 Hz; LF = 0.003–0.2 Hz; HF = 0.2–1 Hz.
- Total power of the FHRV signal, calculated as the sum of the VLF, LF, and HF power;
- Sympahto-vagal balance (SVB), obtained as the ratio between the power in the LF band and the power in the HF band [48].

Six nonlinear indices were also extracted by using the following methodological approaches:

- Sample Entropy (SampEn), which was calculated according to the method proposed by Lake et al. [49];
- Standard deviations perpendicular (SD1) and parallel (SD2) to the line-of-identity calculated through the Poincarè Maps, as described by Fishman et al. [50];
- Higuchi fractal dimension (HFD), calculated according to Higuchi [51];
- Variability Index (VIRR), obtained by applying the Symbolic Dynamic Analysis (SDA), a well-established technique in the analysis of the HRV in adults [52,53], to the series of the difference between consecutive beat-to-beat intervals (ΔRR), in accordance with a methodological approach previously proposed by the authors [54,55,56,57];
- Variability Index (VIFHR) obtained by applying the SDA to the FHRV signal, in accordance with a methodological approach previously proposed by the authors [54,55,56,57].

The whole list of collected, selected, and extracted characteristics of the signal was then reduced to a shortlist of principal features according to a blockwise dimension reduction approach, described in the following paragraph.

### 2.5. Blockwise Dimension Reduction

In order to decrease the number of signal features, without detriment to the clinical meaning they provide as well as to the informative content they can bring, a dimension reduction phase was implemented by adopting a procedure, here called blockwise Principal Component Analysis (bwPCA). In detail, the PCA was applied to blocks of features coming from the same analytic technique. Therefore, the principal components were calculated from time-domain, frequency-domain, and nonlinear FHRV features. The global features, i.e., the pregnancy week and the FHR mean, were kept unchanged since they are of established clinical value. In this way, we attempted to reduce the original set of 19 features to a smaller subset containing meaningful features that take into account different aspects of the examined signal and that can effectively support the interpretation of the results of the study.

The principal components were chosen as those explaining the most variance in the data according to the Kaiser’s rule and varimax criterion in order to compute the fraction of the variables’ total variance explained by each principal component [58], and as those ones whose eigenvalues dominate the eigenvalue spectrum according to the so-called “elbow criterion”, i.e., by choosing those components with eigenvalues higher than 1 (set as the reference threshold) [59]. The correlations between the FHRV features and the principal components were calculated by estimating the Pearson correlation coefficients.

**Figure 4:**
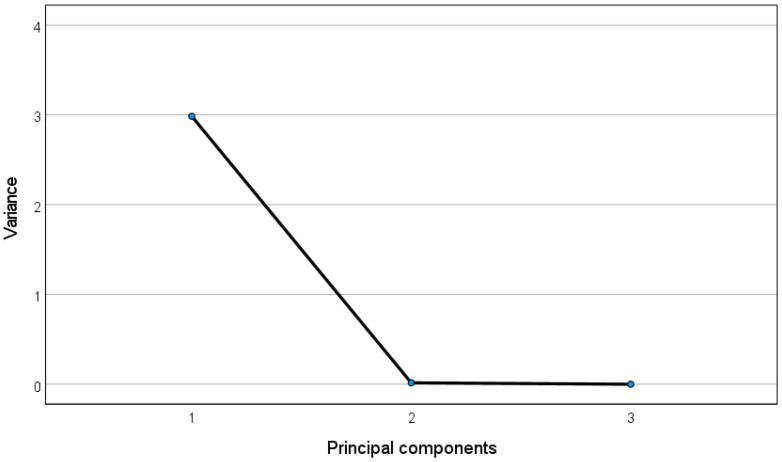
Eigenvalue spectrum for the PCA of the time-domain FHRV features.

In the following, we display the table containing the correlations between the time-domain features and the obtained principal components (Table 1), and the corresponding eigenvalues spectrum (Figure 4), obtained by plotting the eigenvalues of the PCA, which are assumed to equal the variance of the corresponding principal components as described in [60], against the respective principal component.

It can be observed that the first principal component (variance > 1) links well to all the three time-domain features (correlations > 0.99 highlighted in bold).

**Figure 5:**
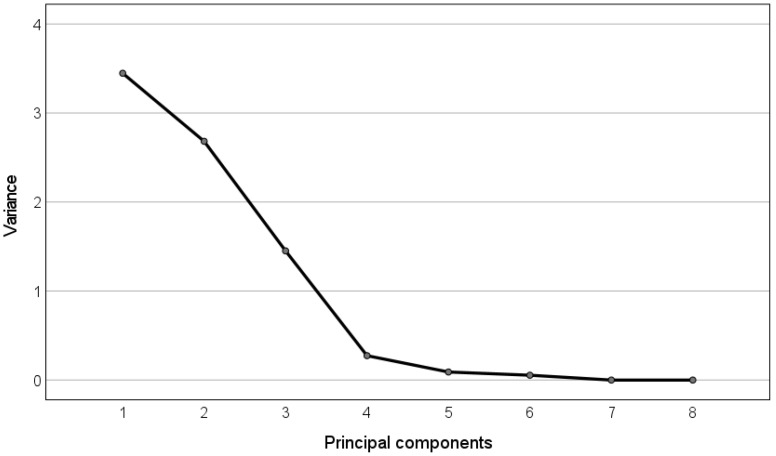
Eigenvalue spectrum for the PCA of the frequency-domain FHRV features.

Analogously, we report in Table 2 the correlations between the frequency-domain features and the obtained principal components, and the corresponding eigenvalues spectrum (Figure 5).

In this case, the first principal component (variance > 3) correlates well with the power in the VLF part of the spectrum and with the Total Power (correlations > 0.80 highlighted in bold). The second principal component (variance > 2) links mainly to the LF and HF indices (correlations > 0.80 highlighted in bold). Finally, the third principal component (variance > 1) links mainly to the SVB index (correlation = 0.94 highlighted in bold).

**Figure 6:**
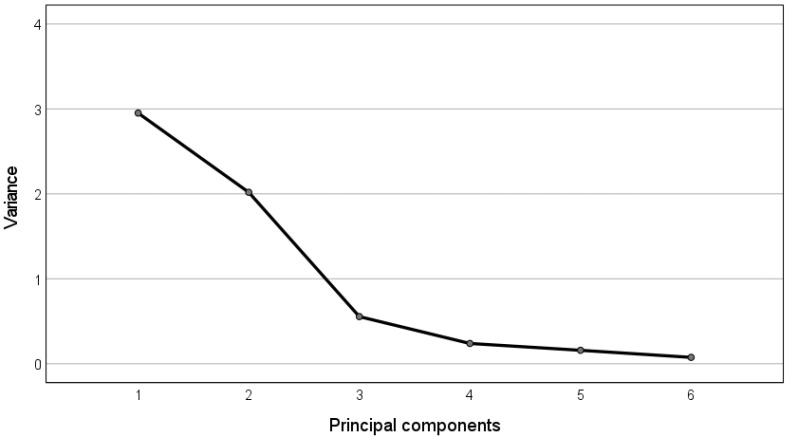
Eigenvalue spectrum for the PCA of the nonlinear FHRV features.

In the following, we report the table containing the correlations between the nonlinear features (Table 3) and the obtained principal components and the corresponding eigenvalues spectrum (Figure 6).

It can be observed that the first principal component (variance > 2) correlates well with the variability indices extracted through Poincarè maps and SDA (correlations > 0.77 highlighted in bold), while the second principal component (variance > 2) links to the complexity indexes, i.e., SampEn and HFD (correlations > 0.86 highlighted in bold).

As a result, the obtained principal components can be recapitulated as follows:

- one time-dependent component, named LIN_time, which is correlated with STV, SDev, and PPA;
- three frequency-dependent components, named LIN_VLF_power, LIN_LF_HF, and LIN_SVB, which are correlated, respectively, with: VLF indices and total power; LF and HF indices; SVB;
- two nonlinear components, named NL_variability and NL_complexity, which are correlated, respectively, with indicators of the variability (SD1, SD2, VIRR, and VIFHR) and complexity metrics (SampEn and HFD).

The summary of the above-mentioned results is reported in Table 4:

The principal components resulting from the application of the bwPCA, together with the two global features that were kept unchanged (pregnancy week and FHR mean), represent a subset of eight meaningful features, i.e., features that can be related to peculiar and interpretable information brought by the examined signals. Indeed, the following characteristics of the signals are represented in the obtained subset: one time-domain information (LIN_time), which represents a statistical description of the variability of the signal [44,45,61,62]; three types of frequency-domain information, i.e., LIN_VLF_power, LIN_LF_HF, and LIN_SVB, respectively, reflecting long period events like thermoregulation (LIN_VLF_power), neural sympathetic activity and fetal breathing (LIN_LF_HF), and the autonomic balance derived from sympathetic and parasympathetic control mechanisms (LIN_SVB) [23,45,63,64,65]; two types of indicators of nonlinear dynamics, the former, NL_variability, mainly related to the intrinsic variability of the FHRV, while the second one, NL_complexity, mainly revealing complex behavior and self-similarities in the time series. Finally, there are two general descriptors of the development and growth of the fetus, indicated by the pregnancy week and the mean FHR.

Such subset of eight meaningful features was used as input to the ANN regression model, whose configuration is described in the following paragraph, in order to improve the interpretability of the results.

### 2.6. Regression by Means of Artificial Neural Network

A multilayer feed forward-back propagation neural network was used in order to model the complex relationships between the FHR signals features and the number of accelerations detected by visual assessment of the signals included in the study. The adopted ANN architecture consists of three separate layers, one input layer, one hidden layer, and one output layer. ANN characteristics and hyperparameters (namely the number of neurons, the transfer functions, and the number of epochs) were tuned in order to select the optimal configuration in terms of predictability. The number of hidden layers was kept to one, in order to keep the network general and avoid overfitting the data. The optimum number of neurons in the hidden layer was selected by testing networks ranging from one to 10 nodes. The best results were obtained from a layer with 10 nodes.

The dataset was randomly divided into training, test, and validation subsets with a ratio of 70:15:15. The target outputs, i.e., the number of FHR accelerations, were not normalized and they ranged from zero to 30 (mean = 8.5; standard deviation = 5.4; median = 8; mode = 9).

### 2.7. ANN Regression Performance Assessment

The predictive capability of the trained model was evaluated by computing the squared correlation coefficient (or determination coefficient, R^2^) against the validation data set, with better predictability indicated by values closer to one. For the proposed model, the correlation coefficient (R), together with R^2^, and the root-mean-square-error (RMSE) were calculated in each subset, as well as in the whole dataset. In particular, the R^2^ and the RMSE were calculated using the following equations

(1) $$\mathrm{RMSE}={\left(\frac{1}{n}\overset{n}{\underset{i=1}{\sum }}{\left(y_{i}-y_{p}\right)}^{2}\right)}^{1/2}$$

(2) $$R^{2}=1-\frac{\sum_{i=1}^{n}{\left(y_{i}-y_{p}\right)}^{2}}{\sum_{i=1}^{n}{\left(y_{i}-{\tilde{y}}_{i}\right)}^{2}}$$

where the numbers y_i_ represent the experimentally observed values, y_p_ are the predicted values, and ỹ_i_ is the mean of y_i_. The closer to one is the R^2^ coefficient, the better is the predictability of the model.

Moreover, the impact of the input variables on the output is determined calculating the contribution of the i-th input data to the k-th output (C_ik_), which represents the relative importance of the input data according to the weightings of the ANN model, by using the following equation

(3) $$C_{\mathrm{ik}}\left|\overset{}{\underset{j}{\sum }}a_{\mathrm{ij}}\times b_{\mathrm{jk}}\right|$$

where the coefficient a_ij_ represents the weighting of the connecting link between the i-th input unit and the j-th hidden unit, while the coefficient b_jk_ is the weighting of the connecting link between the j-th hidden unit and the k-th output unit.

### 2.8. Interpretability and Validity of the Model

Finally, a semi-quantitative method was adopted to get insight into the relationships between the number of accelerations and the nonlinear FHRV indices at different values of the pregnancy week and of the FHR mean. The method involves the use of 3D-graph investigations, obtained from the optimized ANN, showing changes of the output, i.e., the number of FHR accelerations, against two input variables, while keeping the remaining inputs constant at their median values.

As an additional step, in the interpretation and validation of the results obtained with the ANN regression, the model was compared with a multiple linear regression model (MLRM), where the relationship between the predictors and the output was assumed to be linear, according to the following law

(4) $$y=\beta_{0}+\beta_{1}x_{1}+\beta_{2}x_{2}+\beta_{3}x_{3}+\beta_{4}x_{4}+\beta_{5}x_{5}+\beta_{6}x_{6}+\beta_{7}x_{7}+\beta_{8}x_{8}+\varepsilon$$

where y represents the output, x_i_ the i-th independent variable, β_i_ the i-th regression coefficient, and ε the error of the model. The MLRM model was derived assuming a linear dependence between the output and each of the input variables. In addition, the following assumptions were checked before applying the MLRM: independence of the residuals, checked by using the Durbin Watson test; absence of collinearity, checked by calculating Tolerance and Variance Inflation Factor for each variable; normality distribution of the residuals, checked through a Probability–Probability plot; homoscedasticity, checked by plotting the standardized residuals against the standardized predicted values of the model. The critical *p*-value, to state a statistically significant difference, was set at 0.05. The MLRM was estimated to understand how much the changes in the number of FHR accelerations are determined by the selected features.

The two models, ANN regression and MLRM, were compared in terms of their accuracy, measured by the overall R^2^, and of the predictors’ relevance; the latter is measured by the previously described feature importance in the ANN regression model, and by the *p*-value obtained for the independent variables in the MLRM.

## 3. Results and Discussion

**Figure 7:**
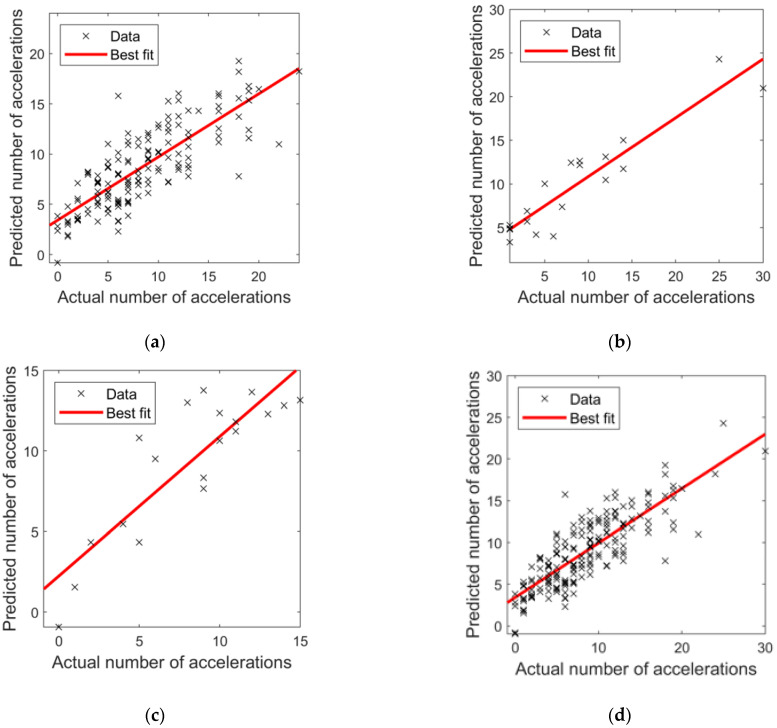
Scatter plots of the number of actual versus predicted accelerations for (**a**) the training subset; (**b**) the validation subset; (**c**) the test subset; (**d**) all data sets. The fitting line (solid) is also reported for each plot along with the identity (X = Y) line (dashed), used as a reference to represent the perfect agreement between real and predicted data.

In Figure 7, the results from the implementation of the ANN regression model are shown by plotting the predicted number of FHR accelerations against the actual number of accelerations for both the training, test, validation, and overall dataset.

It can be observed that the best predictive model obtained R^2^ and RMSE values of 0.65 and 3.09, 0.79 and 3.55, 0.65 and 2.51 for training, validation, and test data, respectively. The overall R^2^ for the model was 0.68 with a RMSE of 3.08, representing a measure of the quality of the trained network and of its moderately high predictability.

**Figure 8:**
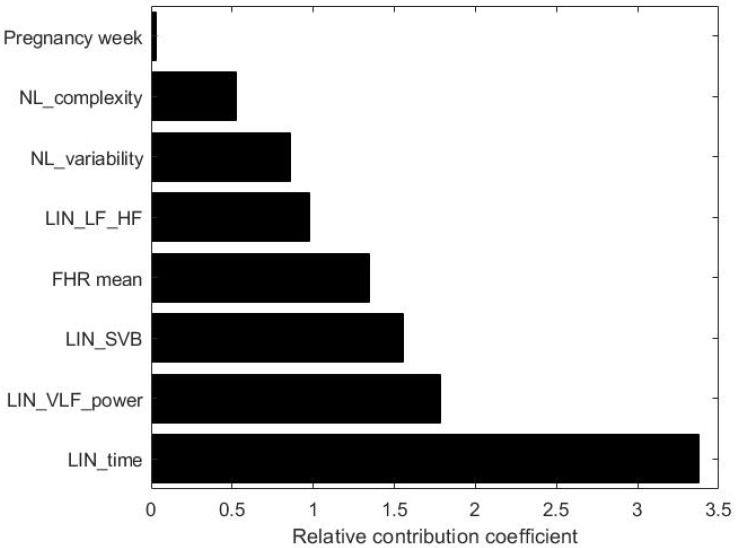
Plot of the ANN feature importance.

In order to estimate which parameter contributes the most, we calculated the relative contribution coefficient for the proposed ANN model (see Figure 8).

As shown in Figure 8, the parameters having the strongest impact are the time-domain features, SVB, VLF, and total power. It is worth noting that the proposed methodology also highlights the relevant contribution of the VLF, which is a generally unexplored factor due to the need for longer signals and due to the fact that the underlying physiological mechanisms are still not fully clear to the medical and scientifical community. On the other hand, nonlinear features and FHR mean give moderate contribution, while the smallest contribution is given by the pregnancy week.

**Figure 9:**
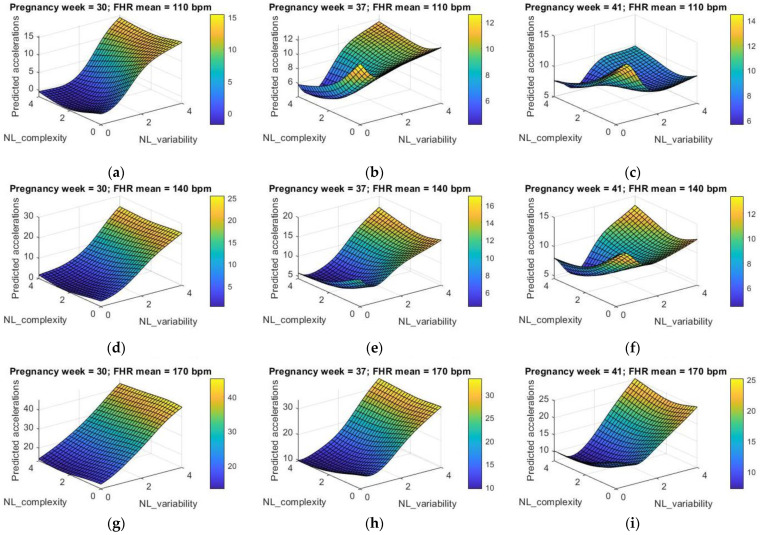
Effect of nonlinear features on the number of predicted FHR accelerations at three different pregnancy periods and three different values of the FHR mean: (**a**) 30th pregnancy week and FHR mean equal to 110 bpm; (**b**) 37th pregnancy week and FHR mean equal to 110 bpm; (**c**) 41st pregnancy week and FHR mean equal to 110 bpm; (**d**) 30th pregnancy week and FHR mean equal to 140 bpm; (**e**) 37th pregnancy week and FHR mean equal to 140 bpm; (**f**) 41st pregnancy week and FHR mean equal to 140 bpm; (**g**) 30th pregnancy week and FHR mean equal to 170 bpm; (**h**) 37th pregnancy week and FHR mean equal to 170 bpm; (**i**) 41st pregnancy week and FHR mean equal to 170 bpm.

In order to evaluate the effect of the nonlinear indices on the number of FHR accelerations, we varied the features NL_variability and NL_complexity, while keeping fixed the other indices to median values, and varying both the FHR mean and the pregnancy week in a three-values range; in particular, we considered low (110 bpm), medium (140 bpm), and high (170 bpm) FHR mean, and middle-early (30th week), middle-late (37th week), and late (41st week) pregnancy period. The results are shown in Figure 9 as three-dimensional graphs.

The impact of nonlinearity of the input–output relationship emerges clearly, with higher degree of nonlinearity at increasing pregnancy week, thus suggesting the more complex dynamics involved in the development of fetuses at term. When the pregnancy week is equal to 30, the variability indices play a major role in determining the fetal activity, with an almost linear tendency showing an increasing number of accelerations at higher values of the variability parameters. The contribution from the complexity indicators becomes more significant at increasing pregnancy weeks; nonlinear dynamics appear at the 37th pregnancy week, showing a stronger competition with the variability indices at a more advanced pregnancy period, as shown by the 3D plot at the 41st week. This is particularly true at an average FHR of 140 bpm or lower (110 bpm), while at higher values of the FHR mean (170 bpm) it can be observed that nonlinearities are less evident, thus suggesting that: (i) signals that are further from healthy conditions (e.g., in the case of a tachycardic tracing, e.g., with high FHR mean) show less complexity than healthy traces, as also confirmed in the literature [66]; (ii) the FHR mean can affect the nonlinear dynamics in the control of the FHRV.

Furthermore, as an additional step in the validation of the ANN regression model, we compared it with the less complex model illustrated in Section 2.7. In particular, if a simple linear relationship between the acceleration of the fetal heart rhythm and the FHRV features is assumed, we derive the MLRM described in Table 5 and Table 6, which also show the results obtained from the application of the MLRM to the examined dataset; the goal is to compare this MLRM with the more sophisticated and general model obtained via the ANN approach.

In Table 5, we report the R^2^ coefficient together with the error of the model, while in Table 6 we show the standardized coefficients obtained by applying the MLRM with their *p*-values and upper and lower bounds of the confidence interval. Statistically significant coefficients are highlighted in bold within the table.

By comparing the results obtained with both the ANN model and the MLRM, we can confirm that the complexity offered by the ANN leads to higher R^2^ values, thus providing more accurate prediction and, thereby, a more promising tool for the investigation of the FHR dynamics. In addition, the higher accuracy of the ANN model strengthens the hypothesis that the relationships existing between the intrinsic characteristics of the FHR signal, here represented by the selected and extracted features, and the accelerations of the fetal heart rhythm, cannot be adequately modeled by using a simple linear regression, but a more complex framework needs to be implemented to model such control mechanisms and their inter-dependency.

Furthermore, by looking at the most influential predictors in both models, it can be observed that the time domain features and the power in the VLF band are both statistically significant in the MLRM and high importance factors in the ANN model. This strengthens the role that such factors have in determining the FHR accelerations. On the contrary, it is worth observing that, despite the lower importance in the ANN model, the complexity-related predictor (NL_complexity) is still statistically significant in the MLRM.

Finally, taken as a whole, the above-mentioned observations could also suggest that, although the FHR mean and the pregnancy week proved to have a lower impact in both the ANN regression model and MLRM, they could still affect the relationships between the FHR accelerations and the nonlinear indices if they are taken into consideration in significantly different time periods and FHR values. Indeed, as shown in Figure 9, the impact of both the pregnancy week and the FHR mean emerges by comparing the dynamics predicted at 110 bpm with those ones predicted at 170 bpm or comparing the predicted dynamics at the 30th week with those ones at the 41st week. In light of these considerations, we could hypothesize that the investigation of nonlinear dynamics as well as the use of predictive tools, such as the ANN, in the field of FHRV should be undertaken carefully and possibly analyzed and interpreted within a limited window, i.e., within a limited pregnancy period or within a limited range of the FHR mean, in order to provide more robust results and reliable information to both clinicians and researchers.

## 4. Conclusions

In this study we showed that ANN proved to be a promising tool to get insight into the relationships between the certain characteristics of the FHR signals (in our case represented by the number of FHR accelerations) and the linear and nonlinear FHRV indices. The obtained results suggest that nonlinear dynamics can also have an impact on the control of FHR accelerations in healthy fetuses, and this is far more evident when the dynamics are compared at largely different ranges of FHR mean and pregnancy periods. The proposed approach makes use of informative and meaningful features obtained by applying a blockwise dimension reduction methodology that helped in the readability and interpretability of the results obtained with the ANN model, which were also compared with a simpler MLRM.

The methodology helped us in studying the accelerations in the FHR traces of healthy fetuses as a function of an ensemble of both traditional and less-conventional FHRV parameters; however, further studies could pursue the objective of investigating also the FHR decelerations in both healthy and pathological fetuses from larger datasets, by taking into consideration specific FHRV indices in order to evaluate, and possibly quantify, the trade-off between linear and nonlinear contributions to the control of FHRV along the course of pregnancy. Moreover, other influential parameters that could be taken into consideration in future investigation include fetal movements, which are associated with accelerations of the heart beat and this correlation between the two is known to increase along the course of pregnancy as widely documented in the literature [67,68,69,70]; fetal behavioral states, which were not included in this study since their definition is dependent on several additional aspects (e.g., eye movements, body movements) [71,72] fall outside the scope of this work; and uterine activity [73], which was not taken into account in the present work since the impact of uterine contraction is mostly influential on the number and type of decelerations rather than accelerations of the FHR; additional features related to FHR acceleration are extracted by applying other techniques, such as the phase-rectified signal averaging [74] and different symbolic dynamics approaches [75].

Further developments of this study will also aim at investigating the role of nonlinear indices in risky or pathological pregnancies. Indeed, as was observed in this work, nonlinearities cannot be neglected in the modeling of FHRV as confirmed by the higher accuracy obtained by adopting the ANN model compared to the MLRM. Despite that, even if a complex ANN model is adopted, traditional time- and frequency-domain features proved to be highly influential parameters in the modeling of HRV in healthy fetuses. This is probably due to the fact that nonlinear dynamics could be even more difficult to understand and interpret and therefore they should be more deeply studied by comparing healthy with pathological conditions rather than focusing only on physiological ones. Therefore, this could suggest that the application of the proposed approach to non-healthy cases would cause changes in the features’ ranking, possibly with variations in the weights attributed to those FHRV parameters highlighting nonlinear dynamics (here identified as variability and complexity components of the FHRV, namely NL_variability and NL_complexity) with respect to linear indices such as time- and frequency-domain ones.

## Figures and Tables

**Table 1:** Dimension reduction of time-domain features of the FHRV.

Time-Domain FHRV Features	Principal Component
	1
STV	**0.995**
SDev	**0.999**
PPA	**0.999**

Correlations > 0.99 highlighted in bold.

**Table 2:** Dimension reduction of frequency-domain features of the FHRV.

Frequency-Domain Features	Principal Components		
	1	2	3
VLF	**0.903**	0.345	−0.013
LF	0.238	**0.932**	0.132
HF	0.275	**0.802**	−0.443
Total power	**0.862**	0.465	0.001
SVB	−0.085	0.286	**0.939**
%VLF	**0.809**	−0.566	−0.089
%LF	−0.788	0.573	0.172
%HF	0.238	**0.932**	0.132

Correlations > 0.80 highlighted in bold.

**Table 3:** Dimension reduction of nonlinear features of the FHRV.

Nonlinear Features	Principal Components	
	1	2
SampEn	−0.165	**0.929**
SD1	**0.774**	0.506
SD2	**0.857**	−0.256
HFD	−0.302	**0.861**
VIRR	**0.873**	0.304
VIFHR	**0.860**	−0.028

Correlations > 0.77 highlighted in bold.

**Table 4:** Final subset of features obtained through the bwPCA.

Type of Feature	Feature	Principal Components
Time domain	STV	LIN_time ^1^
	SDev	
	PPA	
Frequency domain	VLF	LIN_VLF_power ^2^
	LF	LIN_LF_HF ^3^
	HF	LIN_SVB ^4^
	Total power	
	%VLF	
	%LF	
	%HF	
	SVB	
Nonlinear	SampEn	NL_variability ^5^
	SD1	NL_complexity ^6^
	SD2	
	HFD	
	VIRR	
	VIFHR	

^1^ Correlates with STV, SDev, and PPA. ^2^ Correlates with power and its percentage in VLF and with Total power. ^3^ Correlates with power and its percentage in LF and HF. ^4^ Correlates with SVB. ^5^ Correlates with SD1, SD2, VIRR, and VIFHR. ^6^ Correlates with SampEn and HFD.

**Table 5:** Summary of the performance of the MLRM.

R	R^2^	R^2^-Adjusted	Error
0.742	0.551	0.531	3.724

**Table 6:** Coefficient of the MLRM.

Independent Variable	Standardized Coefficient	*p*-Value	Confidence Interval (CI = 95%)	
			Upper CI	Lower CI
intercept	-	0.445	−21.941	9.668
meanFHR	0.126	0.074	−0.008	0.159
Pregnancy week	0.047	0.399	−0.142	0.355
LIN_time	0.499	**0.045**	0.066	5.360
LIN_VLF_power	−0.317	**0.000**	−2.677	−0.776
LIN_LF_HF	−0.060	0.752	−2.369	1.714
LIN_SVB	−0.116	0.175	−1.543	0.282
NL_variability	0.267	0.061	−0.071	2.975
NL_complexity	−0.179	**0.024**	−1.821	−0.127

Statistically significant coefficients are highlighted in bold within the table.

## Data Availability

The data presented in this study are available on request from the corresponding author.

## Abbreviations

ANN: Artificial Neural Network; CTG: CardioTocoGraphy; FHR: Fetal Heart Rate; FHRV: Fetal Heart Rate Variability; HF: High Frequency; HFD: Higuchi Fractal Dimension; HRV: Heart Rate Variability; LF: Low Frequency; MLRM: Multiple Linear Regression Model; bwPCA: blockwise Principal Component Analysis; PPA: Peak to Peak Amplitude; RMSE: Root Mean Square Error; SampEn: Sample Entropy; SDA: Symbolic Dynamics Analysis; SD1: Standard Deviation 1 perpendicular to the line-of-identity (Poincaré maps); SD2: Standard Deviation 2 along the line-of-identity (Poincaré maps); SDev: Standard Deviation; STV: Short Term Variability; SVB: Simpatho-Vagal Balance; VIFHR: Variability Index calculated by applying the SDA to the FHR signal; VIRR: Variability Index calculated by applying the SDA to the series of the difference between consecutive interbeat interval; VLF: Very Low Frequency.
